# CIRSE Standards of Practice on Portal Vein Embolization and Double Vein Embolization/Liver Venous Deprivation

**DOI:** 10.1007/s00270-024-03743-8

**Published:** 2024-06-17

**Authors:** Tiago Bilhim, Georg Böning, Boris Guiu, José Hugo Luz, Alban Denys

**Affiliations:** 1 Interventional Radiology Unit, Curry Cabral Hospital, Unidade Local de Saúde São José; Centro Clínico Académico de Lisboa, SAMS Hospital, Lisbon, Portugal; 2 Department of Radiology, Charité – Universitätsmedizin Berlin, corporate member of Freie Universität Berlin and Humboldt-Universität zu Berlin, Augustenburger Platz 1, 13353 Berlin, Germany; 3 Department of Radiology, St-Eloi University Hospital, Montpellier, France; 4 Department of Interventional Radiology, Brazilian National Cancer Institute (INCA), Rio de Janeiro, Brazil; 5 Department of Radiology and Interventional Radiology, Centre Hospitalier Universitaire Vaudois CHUV, University of Lausanne, Lausanne, Switzerland

**Keywords:** Portal vein embolization (PVE), Liver venous deprivation (LVD), Double-vein embolization (DVE), Post-hepatectomy liver failure (PHLF), Future liver remnant (FLR), Hepatic vein embolization (HVE)

## Abstract

This CIRSE Standards of Practice document is aimed at interventional radiologists and provides best practices for performing liver regeneration therapies prior to major hepatectomies, including portal vein embolization, double vein embolization and liver venous deprivation. It has been developed by an expert writing group under the guidance of the CIRSE Standards of Practice Committee. It encompasses all clinical and technical details required to perform liver regeneration therapies, revising the indications, contra-indications, outcome measures assessed, technique and expected outcomes.

## Introduction

The CIRSE Standards of Practice (SOP) Committee established a writing group which was tasked with producing up-to-date recommendations for performing portal vein embolization (PVE), with or without hepatic vein embolization (HVE). CIRSE SOP documents are not clinical practice guidelines or systematic reviews of the literature. CIRSE SOP documents are not intended to impose a standard of clinical patient care but recommend a reasonable approach to and best practices for performing PVE, with or without HVE. Institutions should regularly review their internal procedures for development and improvement, taking into account international guidance, local resources and regular internal morbidity and mortality reviews.

## Methods

The writing group, which was established by the CIRSE SOP Committee, consisted of 5 clinicians with internationally recognised expertise in PVE. The current document updates the 2010 CIRSE SOP on PVE [1]. A summary of definitions and key recommendations on PVE, sequential PVE followed by HVE, double vein embolization (DVE or bi-embolization or PVE/HVE) and liver venous deprivation (LVD) can be found in Table 1. The writing group reviewed existing literature on PVE/HVE/DVE/LVD, performing a pragmatic evidence search using PubMed to select relevant publications in the English language and involving human subjects, preferably published from 2010 (last SOP document) to date. The final recommendations were formulated through consensus.

**Table 1:** Definitions and key recommendations from the working group on portal vein embolization (PVE), sequential PVE followed by hepatic vein embolization (HVE), double-vein embolization (DVE) or bi-embolization and liver venous deprivation (LVD)

	Definitions	Advantages	Disadvantages	Key recommendations	Expected outcomes
PVE	Selective embolization of portal vein branches	Safe and effective to induce FLR hypertrophy; most widely used technique	May need 2–4 or more weeks to achieve enough FLR hypertrophy; May not induce enough FLR hypertrophy	Should be the first-line approach to induce FLR hypertrophy prior to major hepatectomies; NBCA is the first-line embolic choice	Surgical dropout rates of 15–20% due to disease progression and 5–10% due to insufficient FLR hypertrophy
Sequential PVE followed by hepatic vein embolization	Embolization of the hepatic vein(s) after PVE	Safe and effective to induce FLR hypertrophy	Limited FLR hypertrophy that needs an extra surgical delay of 2–4 weeks after PVE	Should be considered when FLR growth was not enough 2–4 weeks after PVE; usually from a transjugular approach using plugs ± coils ± NBCA	Enhances FLR hypertrophy ± by 10%
DVE or bi-embolization or PVE/HVE	Selective embolization of the portal and hepatic vein branches on the same side of the liver during the same procedure	Potential for faster and more robust FLR hypertrophy with lower rates of surgical dropouts when compared to PVE alone; Potential for lower rates of PHLF	More time-consuming; more expensive; more radiation exposure; potential for more adverse events when compared to PVE alone	Can be considered when very robust and fast FLR hypertrophy is needed as in patients with underlying liver disease or biliary obstruction and very aggressive surgeries (extended right hepatectomy; trissectionectomy); plugs are used to embolize the hepatic vein(s) from a transjugular or transhepatic approach	Surgical dropout rates of 3–36% due to disease progression
LVD	Selective embolization of the portal and hepatic vein branches on the same side of the liver during the same procedure (extended LVD when both lateral and middle hepatic veins are embolized)	Potential for faster and more robust FLR hypertrophy with lower rates of surgical dropouts when compared to PVE alone; Potential for lower rates of PHLF	More time-consuming; more expensive; more radiation exposure; potential for more adverse events; potential risk of non-target NBCA embolization to the lungs; potential for higher intra-operative blood loss and need for transfusions when compared to PVE alone	Can be considered when very robust and fast FLR hypertrophy is needed as in patients with underlying liver disease or biliary obstruction and very aggressive surgeries (extended right hepatectomy; trissectionectomy); Plugs + NBCA are used to embolize the hepatic vein(s) from a transjugular or transhepatic approach	Surgical dropout rates of 3–36% due to disease progression

*PVE* Portal vein embolization; *DVE* Double-vein embolization; *LVD* Liver venous deprivation; *FLR* Future liver remnant; *NBCA* *n*-butyl cyanoacrylate; *PHLF* Post-hepatectomy liver failure

## Background

### Rationale: Post-Hepatectomy Liver Failure

Liver resection is considered the standard of care curative option in patients with secondary or primary liver tumours. Liver surgery for liver tumours is split between liver-sparing surgery, where tumours are resected with oncological margins (tumourectomy), and anatomical resection where the segment or lobe bearing the tumour is resected entirely. Anatomical liver resection is considered a major resection when more than three segments are resected in one surgery [2]. Despite refinements in surgical techniques and improvement in patient preparation and critical care, major hepatectomy still carries a significant risk of morbidity and mortality mainly due to post-hepatectomy liver failure (PHLF).

Reported incidence of PHLF varies widely between 1.2% and 32% due to variations in patient population and procedures, but also due to the lack of a universally accepted definition of PHLF [3]. The International Study Group of Liver Surgery (ISGLS) redefined PHLF based on bilirubin and international normalized ratio (INR) values measured at day 5 or thereafter and proposed a severity grading system [3]. Risk factors for PHLF are related to the volume of future liver remnant (FLR) [4], and also to the underlying liver status such as post-chemotherapy liver injury [5, 6] or chronic liver disease such as cirrhosis [7] or chronic cholestasis [8]. Recently, in complement to the FLR volume, FLR function appeared as an important factor associated with PHLF [9, 10]. To identify patients with insufficient liver function in the FLR, new nuclear medicine investigations have been developed such as mebrofenin scintigraphy [8, 9] allowing quantification of liver function.

### Strategies to Increase the Volume of the Future Liver Remnant

Strategies to increase the FLR volume preoperatively have been developed over the years, allowing more patients to undergo successful surgical resection. The first is PVE, which consists of embolizing the portal branches of the future resected liver (i.e. usually right liver portal branches) 3–5 weeks before hepatectomy [11]. This method has a very low morbidity and is well tolerated [12] and carries a high success rate. However, in 15–20% of patients after PVE, the planned hepatectomy cannot be performed either due to tumour progression or due to insufficient hypertrophy [13]. There is a surgical equivalent technique which is portal vein ligation that can sometimes be used, especially when tumourectomies are to be performed on the FLR. With this surgery, one of the portal vein branches is ligated and can also be embolized with ethanol. Another option is to perform ablation of the FLR tumours and PVE at the same time. To reduce the risk of tumour progression, by decreasing the delay between liver preparation and resection, an aggressive surgical approach has been developed associating right portal vein ligation and liver partition—so called ALPPS (associating liver partition and portal vein ligation for staged hepatectomy) one week before hepatectomy [14]. Despite initial enthusiasm due to very rapid FLR hypertrophy, concerns were raised due to the high morbidity and mortality rates seen with this surgical strategy [15]. One possible explanation for these disappointing results is the fact that despite a rapid volumetric gain, the function of the FLR does not necessarily mirror the volumetric gain and may even decrease [16]. Preoperative FLR functional evaluation has been advised after studies assessing ALPPS series revealing that volumetric gain might not be followed by functional gain [16]. After ALPPS first-stage, liver functional gain was only half of the volumetric gain, explaining the high rate of morbidity and mortality following this regenerative surgical approach [16]. One other strategy has been explored in patients with insufficient liver volume gain four weeks after PVE, by adding HVE. This strategy, called sequential PVE followed by HVE, has shown to add further liver regeneration [17]. A major drawback of this approach is the delay between each procedure increasing the risk of liver or extrahepatic tumour progression. A delay of four weeks is necessary to assess liver volumetric gain after PVE followed by another four weeks after additional HVE [17–19]. In this context, the concept of combining PVE and HVE in the same procedure appeared recently under different descriptions in the literature such as LVD and DVE (i.e. bi-embolization) [20, 21]. The theoretical benefit is to accelerate and induce more hypertrophy than PVE alone and to reduce the risk of absent or insufficient hypertrophy. After PVE, portal vein collaterals between the FLR and the embolized liver develop and compromise FLR hypertrophy. Embolizing ipsilateral hepatic and portal vein branches with ALPPS or LVD/DVE will block the development of these portal vein collaterals between the FLR and the embolized liver. This will optimize flow and minimize hepatotrophic factor washout from the FLR, enhancing hypertrophy [14–21].

### Indications and Contra-Indications for Liver Regeneration Techniques Prior to Major Hepatectomies

Indications for these liver regeneration techniques are based on liver volume assessment by computed tomography (CT). All surgical groups agree that a FLR/TLV (total liver volume) ratio below 20% is an indication for a liver regeneration technique such as PVE, DVE, LVD or ALPPS. Debates about the use of either the functional liver volume measured by CT or the estimated total liver volume based on body surface area are still unresolved [22, 23]. Both methods can be used, but they carry some discrepancies in 5% of patients [24], potentially leading to decision errors regarding PVE indication. Of course, estimating the liver volume by CT does not estimate the function and regenerative capacity of FLR after resection. Many groups also consider the liver function estimated either by mebrofenin scintigraphy-CT or by MRI using hepato-specific contrast agent. These two imaging techniques can estimate hepatocyte function by quantitative imaging [8, 25, 26]. PVE/DVE/LVD can be proposed in patients bearing colorectal (or other less frequent) liver metastases, but also before major hepatectomy for hepatocellular carcinoma, or intrahepatic cholangiocarcinoma or hilar type (Klatskin) cholangiocarcinoma [27, 28]. The threshold of FLR/TLV ratio for PVE or DVE/LVD is different among these different groups and varies between 20% (colorectal metastases), 30% (colorectal metastases with post-chemotherapy liver injury) and 40% (hepatocellular carcinoma with underlying liver cirrhosis and Klatskin tumours) [27]. The suggested thresholds for resections are shown in Table 2. There are no absolute contra-indications for PVE or DVE/LVD if the patient is considered eligible for major hepatectomy. For patients with Klatskin tumours and obstructive jaundice, most centres recommend biliary drainage before liver regeneration treatments and bilirubin levels < 5–10 μmol/L, even though simultaneous biliary drainage and PVE has been shown to be safe and effective [28].

**Table 2:** Definitions and recommended outcome measures assessed after liver hypertrophy techniques

Type of hepatic surgery [2]	Major hepatectomy—resection of 4 or more segments	Right hepatectomy—resection of segments V–VIII; left hepatectomy—resection of segments II–IV (± segment I)	Extended hepatectomy (≥ 5 hepatic segments)—resection of segments V–VIII + I or IV (right)	Trisectionectomy—resection of segments V–VIII + sIV + sI (right); resection of segments I–IV + sVIII and sV (left—also known as extended left hepatectomy)	Left lobectomy—resection of segments II and III
PHLF [3, 68]	ISGLS	50/50 Criteria—concomitant presence of prothrombin time (PT) < 50% and serum total bilirubin > 50 μmol/L on postoperative day 5			
ISGLS PHLF grades [3]	Grade A—deterioration from the preoperative baseline in routine laboratory tests indicating a postoperative impairment of liver function, with no clinical symptoms deviating from a normal, expected postoperative course, not requiring additional diagnostic evaluation, and can be managed on the regular ward	Grade B—there is a deviation from the regular, postoperative clinical pathway, but can be managed without invasive treatment; may need transfer to the intermediate or intensive care unit, which is related directly to the abnormal hepatic function	Grade C—requiring an invasive procedure such as haemodialysis, intubation and mechanical ventilation, extracorporeal liver support, rescue hepatectomy, transplantation and circulatory support (vasoactive drugs)		
FLR hypertrophy assessment [22–24]	FLR/TLV ratio (%) = (FLR volume) × 100/(TLV volume)	FLR/body weight ratio (%) = (FLR volume × 1.19) × 100/body weight (g)	DH = FLR/TLV ratio post PVE/LVD–FLR/TLV ratio pre PVE/LVD	KGR = DH/number of weeks after PVE/LVD	FLRabh (%) = (FLR volume after PVE – FLR volume before PVE) × 100/(FLR volume before PVE)
Functional FLR evaluation [25, 26]	Mebrofenin hepatobiliary scintigraphy—^99m^Tc-mebrofenin uptake /BSA (%/min/m^2^)	ICG fluorescence imaging—retained ICG/all ICG given at 15 min after administration (ICG-R15—mostly used in cirrhotics)	Gadoxetic acid-enhanced MRI—function measured based on the hepatic uptake of hepatospecific MR contrast agents		
Suggested safe thresholds for major hepatectomy [8, 22–26]	FLR/TLV ratio > 20% for normal liver; > 30% for livers with prior chemotherapy; > 40% for cirrhotic or cholestatic livers	FLR/body weight ratio > 0.5 for normal liver; > 0.8 for livers with prior chemotherapy; > 1.4 for cirrhotic or cholestatic livers	KGR > 2	Mebrofenin hepatobiliary scintigraphy uptake > 2.7/min/m^2^ or 8.5%/min	In cirrhotics, ICG retained at 15 min < 10% (normal); may need bigger FLR/TLV ratios (50%) when 10–20%; surgery is contraindicated if > 20%

*PHLF* Post-hepatectomy liver failure; *ISGLS* International study group of liver surgery; *FLR* Future liver remnant; *TLV* Total liver volume; *NBCA* *n*-Butyl cyanoacrylate; *DH* Degree of hypertrophy; *PVE* Portal vein embolization; *LVD* Liver venous deprivation; *KGR* Kinetic growth rate; *FLRabh* FLR absolute hypertrophy; *BSA* Body surface area; *ICG* Indocyanine green; *MRI* Magnetic resonance imaging

## Patient Preparation

The decision for liver regeneration therapies should be made in multidisciplinary team meetings (MDT) of surgeons, hepatologists, oncologists, and interventional radiologists with respect to the individual patient’s performance status, tumour resectability and vascular invasion, FLR volume and function, type of tumour and underlying liver disease. Informed consent should be obtained 24 h ahead of treatment. In addition to routine laboratory parameters, particularly for coagulation and bilirubin, pre-surgical work-up (within the month preceding intervention) should include CT/MRI-based liver volume analysis (FLR/TLV/FLR ratio) and ideally should also include functional liver assessment. If additional ablations are planned within the FLR, the corresponding volumes must be considered in the calculation.

As biliary obstruction can impair liver regeneration and hypertrophy, the biliary tree of the FLR should be drained before PVE/DVE/LVD when bilirubin levels > 2 μmol/L (threshold for a safe major hepatectomy) [28]. For patients with hepatocellular carcinoma with underlying liver cirrhosis, transarterial chemoembolization may be performed 3–4 weeks before PVE/DVE/LVD, enhancing the regeneration of the FLR [29, 30]. The CIRSE SOP on Peri-operative Anticoagulation Management During Interventional Radiology Procedures should be followed for PVE/DVE/LVD [31]. There are guidelines for prophylactic antibiotic therapy in interventional radiology in general but not for PVE/DVE/LVD in particular; therefore, decisions should be made on a case-by-case basis considering individual risk factors (prior biliary manipulation or infection), the patient’s history and the therapeutic strategy including combinations of vascular/non-vascular interventions [32].

## Portal Vein Embolization Technique

After skin disinfection and local anaesthesia, PVE is usually performed via ultrasound-guided percutaneous transhepatic portal vein access using a 21–22G needle and 0.018-inch guide wire included in dedicated tri-axial micropuncture kits (with a metallic stiffening cannula, a dilator, and a 10–20 cm long 4–6 Fr. sheath) [1, 11, 12]. Alternative routes include transileocolic and transsplenic approaches. The transileocolic technique necessitates a mini-laparotomy and can also be part of a two-stage surgical resection. The transhepatic route allows for the procedure to be conducted via either ipsilateral or contralateral puncture. The advantages of ipsilateral (i.e. right access for right PVE) access are: no damage to the FLR, ability to gain access to segment IV portal venous branches and reduced risk of radiation exposure to the operator’s hand. Its drawbacks include the need for reversed catheters that are used for PVE and for the completion portography, with longer and more complex procedures. The contralateral approach may induce damage to the FLR and poses more radiation exposure to the operator, but it provides more direct access to the target portal vein branches [1, 11, 12]. Catheters used are usually 4–5 Fr. reversed catheters for ipsilateral approach and 4–5 Fr. angled-tip catheters for contralateral approach. When the embolic agent used is *n*-butyl cyanoacrylate (NBCA), it is recommended to perform the embolization with 2.4–3.0 Fr. microcatheters. NBCA can adhere to the interior of catheters and migrate to untargeted portal vein branches. Thus, using super selective catheterization with microcatheters reduces the risk of untargeted embolization to portal vein branches of the FLR.

After gaining access to the portal system, 2D (20 ml of contrast medium; 10 ml/s contrast injection; right anterior oblique view of 25°) or 3D portography (35 ml of 1:1 or 3:2 iodine contrast to saline mixture; 5 ml/s contrast injection; with 2 s X-ray delay and 5 s acquisition/rotation time; volume of contrast adjusted to acquisition/rotation time) is recommended to assess the portal anatomy. With ipsilateral approaches, the portography should be performed with reversed catheters and selective embolization of portal vein branches can be performed via the same catheter when particles and coils are used. However, microcatheters should be used for embolization with NBCA. The same reversed catheter can be used for the completion portography. With contralateral approaches the same angled-tip catheter can be used for portography and embolization but a different catheter should be used for completion portography, when NBCA is used. Embolization of segment IV portal venous branches, although technically challenging, is safe in experienced hands and can improve hypertrophy and extend resectability [33] at the expense of a technically challenging procedure (4–6 branches usually supply segment IV). Cone-beam CT or angio-CT technologies with 3D road-mapping and microcatheters are therefore recommended.

Embolic materials used for PVE [34] include absolute ethanol [35–37], polyvinyl alcohol (PVA), gelatin sponge, fibrin glue, NBCA mixed with lipiodol [38], polidocanol-foam, or hybrid combinations of these agents with coils [39] or vascular plugs [40, 41]. Several studies showed a benefit of NBCA over PVA [42] and microparticles + coils [43–45] in terms of FLR hypertrophy, with similar complication rates. For particles, 200–1000 µm particles (spherical or non-spherical) are recommended followed by 8 mm–16 mm diameter coils placed in the portal vein branch being embolized, through 4–5 Fr. catheters. Plugs used are usually 8–18 mm in diameter (10–20% oversizing) and will require larger (6–8 Fr.) sheaths for deployment. NBCA/lipiodol dilutions of 1/4–1/8 are recommended and care must be taken to flush all catheters with dextrose 5% and to avoid saline. Continuous injection of glue or a sandwich technique (injection of small amounts of NBCA interspaced with dextrose flushing) are based on operator preference.

## Double Vein Embolization and Liver Venous Deprivation Technique

The LVD technique was originally outlined as a fully transhepatic procedure [20]. Hepatic veins (HVs)—right, optional accessory right, and possibly middle (extended LVD)—are initially accessed with ultrasound guidance using a 21–22 G Chiba needle and a 0.018-inch wire, along with a micropuncture set. PVE is performed after wire/sheath access to the HVs. A 6–10 Fr. sheath is placed in each HV to deploy a vascular plug, positioning its distal end about 15 mm proximal to its junction with the vena cava. The plug positioning is crucial, so that surgical HV ligation with a stapler is not compromised during the planned hepatectomy. The vascular plug should be oversized by around 40% to prevent migration, and its accurate placement can be confirmed with ultrasound. Veno-venous collaterals quickly develop after proximal occlusion by the vascular plug, which is the reason why glue at a lower dilution (1/3–1/5) has been proposed to occlude not only distal venous branches but also these collaterals. These collaterals pose a challenge as they may lead to untargeted NBCA embolization to the lungs. This is one of the reasons why some centres prefer DVE over LVD. All transhepatic accesses can be embolized with glue, gelfoam or coils. The same LVD technique can be performed via a transjugular or transfemoral approach, with a micro-catheter in parallel to the vascular plug, to inject glue. The critical step is to prepare the glue and iodized oil mixture prior to plug deployment and to start injecting the mixture immediately behind the plug due to the quick expansion of these collaterals when outflow is blocked. Since, the middle HV drains approximately 2/3 of the right paramedian sector, the tolerance of LVD led to a proposal to extend LVD (extended LVD) embolizing not only the right (and accessory right HV when present) but also the middle HV using the exact same technique [46].

The notion of DVE or bi-embolization has been introduced but is specific to deploying only vascular plugs ± coils in the HVs via transjugular or transhepatic access [21]. This method may leave distal collaterals open, which can bypass the vascular plug within minutes. Thus, multiple vascular plugs ± coils should be used to occlude the whole HV. LVD embolizes both proximal (via vascular plug) and distal venous branches (via NBCA), as well as potential collaterals. The use of multiple vascular plugs placed all along the HVs and their tributaries can be considered instead of vascular plug + glue [47]. No data are currently available on the most optimal technique. PVE should be performed prior to DVE or LVD to allow better glue penetration into portal vein branches and reduce the risk of contralateral non-target glue reflux [48].

## Peri-Procedural and Follow-Up Care

PVE/DVE/LVD can be performed in an outpatient setting; however, a short (1-day) inpatient stay is usually preferred. According to the CIRSE SOP on Analgesia and Sedation for Interventional Radiology in Adults, accurate evaluation, monitoring and documentation of patient care is important to minimise the risk of adverse events [49]. Procedural medication and monitoring of the patient should be delivered by a team member other than the specialist performing the procedure [49]. PVE/DVE/LVD can be performed using only pain medication without sedation in most cases. Nevertheless, sedation or general anaesthesia is also possible and might be required when NBCA is used as it induces more inflammation and pain when compared to PVA and coils. Whenever deeper levels of sedation are required, anaesthetists should be involved [49]. Follow-up imaging to assess hypertrophy of the FLR should ideally be performed using the same modality as before the procedure and timed to coincide with the planned surgery, ideally 14–28 days after the intervention. The time from PVE/DVE/LVD to planned hepatectomy varies between teams and clinical scenarios ranging from 2–4 weeks in non-cirrhotics to 2–3 months in cirrhotics [11–21]. Asthenia may develop after LVD, thus routine multivitamin and phosphorus supplementation may be considered, which may also enhance FLR hypertrophy [20, 21, 46, 47].

## Outcomes

### Technical/Clinical Outcomes and Complications

Technical and clinical success after PVE (complete occlusion of all planned portal branches and enough FLR regeneration for the intended hepatectomy, respectively) is very high, and should be expected to be above 98% and 90%, respectively [34, 38]. Common reasons for technical failure are inability to access the portal system (blocked needle pathway due to liver tumour burden or altered portal anatomy caused by the tumour mass or unexpected thrombosis of the portal system) or incomplete embolization of the portal vein branches (missed portal vein branch during PVE) [44, 50]. Lack of the expected FLR regeneration after a technically successful performed PVE, measured by the degree of hypertrophy (DH) and kinetic growth rate (KGR), has been associated with a significantly higher incidence of PHLF [51, 52], and should be interpreted as an alert sign of a poor background liver function and reserve.

One of the most feared complications after PVE is that it may preclude the awaited liver surgery. A systematic review revealed that in 0.4% of cases, patients will not proceed to the planned hepatectomy due to major complications (severe cholangitis, large abscesses and sepsis, and portal venous or mesentericoportal venous thrombosis) [34]. As with other hepatic percutaneous procedures, complications after PVE may arise due to the transhepatic access, such as subcapsular haematoma, haemoperitoneum, pneumothorax, haemobilia, arteriovenous shunts, pseudoaneurysm, cholangitis and sepsis. Other complications such as post-embolization syndrome, non-target embolization, portal vein thrombosis and transient liver failure may also occur. The complication threshold for PVE should be expected to be 2.5% [53]. Mortality after PVE is rare and has been scarcely reported. In a meta-analysis consisting of 1088 patients, the overall morbidity rate for PVE was 2.2% without mortality [53]. In a single-institution large cohort of 146 patients, the thirty-day mortality was 0.7% [38]. Mortality rate after PVE should be expected to be maximum of 0.1% [34].

It has been suggested that PVE might lead to tumour progression and worse oncological outcomes in patients submitted to liver surgery [54, 55]. The arterial buffer phenomenon (increase of liver arterial perfusion after portal vein occlusion) [56] and the release of hypertrophic factors involved in the regeneration process may stimulate tumour growth. Nevertheless, larger studies, including a systematic review with meta-analysis, showed no adverse effect in the local liver tumour progression, nor in the overall survival of patients treated with liver surgery, with versus without prior PVE [57].

### Future Liver Remnant Hypertrophy Outcome Measures

Regenerative results of PVE have been extensively reported. Commonly reported hypertrophy outcome parameters are FLR absolute hypertrophy, DH and KGR. FLR absolute hypertrophy is related to the FLR growth in relation to its initial volume. DH shows the increase in the FLR ratio after PVE. KGR is a dynamic outcome measurement, a time-lapse of the regenerative process. It is calculated by dividing DH by the waiting time after PVE, usually in weeks. KGR shows how fast the regenerative process takes place. KGR ≥ 2 has been directly correlated with the absence of PHLF and lower mortality after major hepatectomies [51]. Highly relevant clinical outcomes such as PHLF, peri-operative transfusion requirements, operative times, and complication rates after hepatectomies in patients submitted to liver regeneration therapies have been reported (Table 3). Patients who develop PHLF show longer intensive care and hospital stays and more postoperative complications (infectious; bile leaks; bleeding) [58, 59]. PHLF developed in 10% and 9% of the patients for the group submitted to major hepatectomies with and without PVE, respectively [59]. The five-year overall survival was similar between these two groups, being 38.2% and 37.9% for patients with and without PVE, respectively [59].

**Table 3:** Expected outcomes after portal vein embolization (PVE), sequential portal vein embolization followed by hepatic vein embolization (HVE), double vein embolization (DVE) and liver venous deprivation (LVD)

	Adverse events	Technical success	Clinical success	Surgical outcomes	Volumetric changes
PVE [27, 34, 39, 42–45, 53, 58, 59]	2.5%	98%	90–95%	PHLF rates 0–13%; blood loss 837 ml and need for transfusions 13–20%, operative times 340 min, and complication rates 5.8–21%	DH 12–16% KGR 3–6 FLRabh 37–57%
Sequential PVE followed by hepatic vein embolization [17]	NR	91.7–100%	84%^b^	PHLF rates, blood loss NR and need for transfusions 44%, operative times NR, and complication rates 11.1%	DH 10% KGR NR FLRabh NR
DVE [21, 48, 60, 67]	NR	NR	> 95%	PHLF rates 0%^a^; blood loss 1310 ml and need for transfusions NR, operative times 419 min, and complication rates 15%	DH 10% KGR 18 FLRabh 51.2%
LVD / eLVD [20, 46, 47, 61–63]	NR	100%	> 95%	PHLF rates 23.1%; blood loss 1200 mL and need for transfusions 7.7%, operative times 330 min, and complication rates 10%	DH 12.7%^c^ KGR 4–24 FLRabh 53%^d^

*PVE* Portal vein embolization; *DVE* Double-vein embolization; *LVD* Liver venous deprivation; *eLVD* Extended LVD; *NR* Not reported; *FLR* Future liver remnant; Clinical success defined as achieving the intended FLR volume and ratio for hepatic resection; *PHLF* Post-hepatectomy liver failure; *DH* Degree of hypertrophy; *KGR* Kinetic growth rate; *FLRabh* FLR absolute hypertrophy

^a^Different criteria for PHLF were used among studies (50–50 criteria [68], ISGLS criteria [69])

^b^Clinical failure for this procedure was reported in cirrhotic cohorts

^c^only reported for LVD and not for eLVD

^d^ After three weeks [61]/53.4% at seven days for eLVD [46]

After PVE, > 70% of patients undergo the planned liver surgery, and this threshold should be considered acceptable [34, 44, 59]. Nevertheless, 20–30% of patients submitted to PVE will not go through the planned hepatectomy, the main reason being disease progression in the waiting period after PVE. Another reason for not performing the planned liver resection is insufficient growth after PVE in 5–10% of patients [34, 44, 59].

### Outcomes for Portal Vein Embolization, Double Vein Embolization and Liver Venous Deprivation

In 2021 two systematic reviews and one randomized trial evaluated the effect of different embolic materials in liver regeneration after PVE, showing the superiority of NBCA over PVA plus coils [44, 45, 58]; the beneficial increment in liver regeneration when segment IV embolization was performed; and the non-influence of neo-adjuvant systemic chemotherapy on PVE regenerative results [44]. In the aforementioned RCT [44], PHLF was 13% for the NBCA group and 27% for the PVA plus coils group (*p* = 0.27).

Different strategies have been proposed to overcome the potential insufficient regeneration after PVE, including sequential HVE after PVE [17], LVD [20], DVE [21] and ALPPS [14]. Sequential HVE has been shown to increase the volume of the FLR after insufficient PVE. The threshold of 5–10% increase in the FLR absolute growth should be expected 2 weeks after hepatic vein embolization in patients already submitted to PVE [17–19]. Interestingly, although sequential hepatic vein embolization is very well tolerated and complication rates are similar to PVE, reports of middle hepatic embolization erroneously recognized and embolized as the right hepatic vein have been reported from different groups [17, 60].

Up to now there were only small retrospective cohorts and systematic reviews comparing the regenerative results of PVE and LVD. The latter technique seems to promote superior regenerative results. One study [47] reported superior KGR and FLR absolute hypertrophy for LVD when compared to PVE. Another study found superior volumetric and functional FLR gain when using LVD compared to PVE [61]. FLR functional gain after LVD was obtained earlier than volumetric hypertrophy [62]. These findings suggest that the planned hepatectomy might be performed (1 or 2 weeks) earlier after LVD when compared to PVE [61, 62]. Interestingly, in these studies, KGR results (1.4%/week) and FLR volume increase (14–19%) for PVE were unusually low, maybe explaining the LVD superiority. Negative studies comparing PVE and LVD have also been reported. A prospective cohort of 14 patients submitted to LVD compared by matched analysis to PVE showed no FLR hypertrophy superiority for LVD before major hepatectomies [63]. Strong evidence about the indications, technique, complications, hypertrophy results and clinical outcomes of LVD and eLVD (extended LVD) as well as DVE will soon be available through ongoing multicentre clinical trials [64, 65]. DVE and LVD have shown similar safety and hypertrophy results, but direct comparative trials are lacking [61, 66, 67].

## Conclusion

**Fig. 1:**
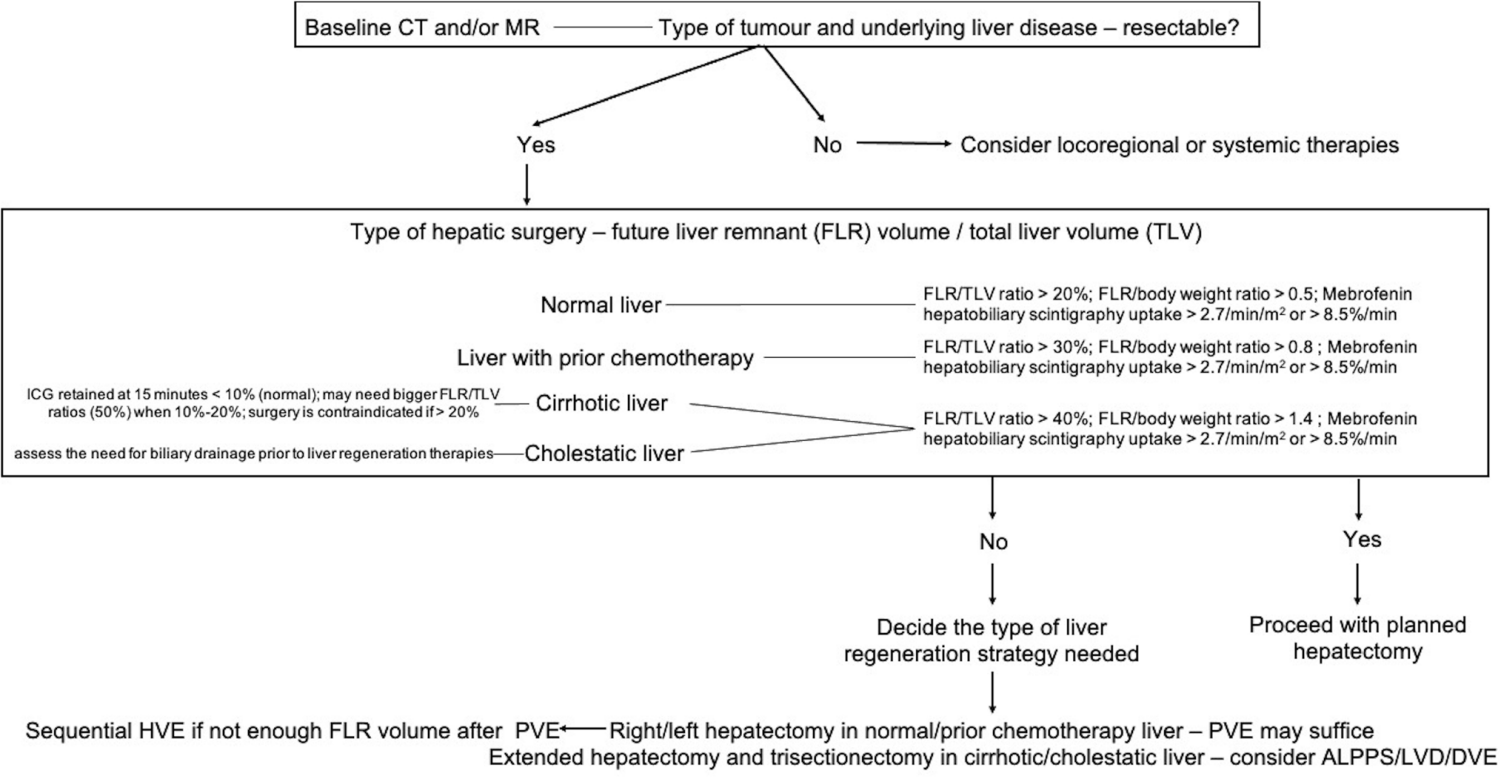
Flowchart of patient assessment prior to major hepatectomy. ICG, Indocyanine green; PVE, Portal vein embolization; ALPPS, Associating liver partition and portal vein ligation for staged hepatectomy; LVD, Liver venous deprivation; DVE, Double-vein embolization; HVE, Hepatic vein embolization

A summary of the working group recommendations can be found in Table 4 and Fig. 1.

**Table 4:** Summary of working group recommendations

Summary of recommendations
**MDT decision**
Type of tumour and underlying liver disease
Type of surgery planned (FLR definition and volumetric/functional analysis)—resectability
Assess the need for biliary drainage prior to liver regeneration therapies
Decide the type of liver regeneration strategy needed
**Pre-procedural work-up**
CT and/or MR imaging of the liver 1 month prior to intervention with FLR definition and volumetric analysis
Coagulation status, bilirubin levels
MDT decision (type of surgery needed)
Informed consent
Antibiotic prophylaxis
**Procedural steps**
Ultrasound guided percutaneous transhepatic access to the portal vein ± hepatic vein(s)
Ipsilateral or contralateral approach for PVE
NBCA as first-line embolic option for PVE
Plugs for hepatic vein embolization (1–2 cm distal to the inferior vena cava) with additional NBCA for LVD (only plugs ± coils in DVE)
Hepatic vein embolization may also be performed via transjugular approach
Embolization of segment IV of portal vein might be needed in specific situations
Hepatic vein embolization (DVE or LVD) should be performed after PVE completion [48]
DVE/LVD increase procedural times and costs and may interfere with planned surgery (increased bleeding, difficulty in HV ligation with a stapler when the plug is positioned close to the inferior vena cava)
**Post-procedural management**
Pain management and multivitamin/phosphorus supplements after PVE/LVD
Volumetric and functional liver assessment with CT/MR and scintigraphy 14–28 days after intervention
Correct timing for planned surgery

*MDT* Multidisciplinary team meeting; *FLR* Future liver remnant; *PVE* Portal vein embolization; *NBCA* *n*-Butyl cyanoacrylate; *LVD* Liver venous deprivation; *DVE* Double-vein embolization

## Abbreviations

SOP: Standards of Practice
PVE: Portal vein embolization
LVD: Liver venous deprivation
DVE: Double-vein embolization
HVE: Hepatic vein embolization
PHLF: Post-hepatectomy liver failure
ALPPS: Associating liver partition and portal vein ligation for staged hepatectomy
FLR: Future liver remnant
TLV: Total liver volume
MDT: Multidisciplinary team meeting
NBCA: *n*-Butyl cyanoacrylate
PVA: Polyvinyl alcohol
DH: Degree of hypertrophy
KGR: Kinetic growth rate
eLVD: Extended liver venous deprivation
